# ALG13 participates in epileptogenesis via regulation of GABA_A_ receptors in mouse models

**DOI:** 10.1038/s41420-020-00319-6

**Published:** 2020-09-17

**Authors:** Junming Huo, Shuanglai Ren, Peng Gao, Ding Wan, Shikuo Rong, Xinxiao Li, Shenhai Liu, Siying Xu, Kuisheng Sun, Baorui Guo, Peng Wang, Baoli Yu, Ji Wu, Feng Wang, Tao Sun

**Affiliations:** 1 Ningxia Key Laboratory of Cerebrocranial Diseases, Ningxia Medical University, 1160 Shengli Street, Yinchuan, 750001 Ningxia China; 2 Department of Neurosurgery, General Hospital of Ningxia Medical University, 804 Shengli Street, Yinchuan, 750001 Ningxia China; 3 Renji Hospital Shanghai Jiaotong University School of Medicine, Key Laboratory for the Genetics of Developmental and Neuropsychiatric Disorders (Ministry of Education), Bio-X Institutes, Shanghai Jiao Tong University, Shanghai, 200240 China; 4 Ningxia Key Laboratory of Reproduction and Genetics, Ningxia Medical University, 1160 Shengli Street, Yinchuan, 750001 Ningxia China

**Keywords:** Epilepsy, Epilepsy

## Abstract

ALG13 (asparagine-linked glycosylation 13) plays crucial roles in the process of N-linked glycosylation. Mutations of the ALG13 gene underlie congenital disorders of glycosylation type I (CDG-I), a rare human genetic disorder with defective glycosylation. Epilepsy is commonly observed in congenital disorders of glycosylation type I (CDG-I). In our study, we found that about 20% of adult ALG13KO knockout mice display spontaneous seizures, which were identified in a simultaneous video and intracranial EEG recording. However, the mechanisms of ALG13 by which deficiency leads to epilepsy are unknown. Whole-cell patch-clamp recordings demonstrated that ALG13KO mice show a marked decrease in gamma-aminobutyric acid A receptor (GABA_A_R)-mediated inhibitory synaptic transmission. Furthermore, treatment with low-dose diazepam (a positive allosteric modulator of GABA_A_ receptors), which enhances GABA_A_R function, also markedly ameliorates severity of epileptic seizures in ALG13KO mice. Moreover, ALG13 may influenced the expression of GABA_A_Rα2 membrane and total protein by changing transcription level of GABA_A_Rα2. Furthermore, protein interactions between ALG13 and GABA_A_Rα2 were observed in the cortex of wild-type mice. Overall, these results reveal that ALG13 may be involved in the occurrence of epilepsy through the regulation of GABA_A_R function, and may provide new insight into epilepsy prevention and treatment.

## Introduction

Epilepsy is one of the most common neurological disorders that affects 1–2% of the population worldwide across different ages and background. Epilepsies are characterized by recurrent seizures and are caused by the imbalance of excitatory and inhibitory neural circuits^1,2^. Asparagine-linked glycosylation 13 (ALG13) is located on the X-chromosome and encodes a protein that heterodimerizes with ALG14 to form a functional UDP-GlcNAc glycosyltransferase in the endoplasmic reticulum that catalyzes the second step of protein N-glycosylation^3,4^. Protein asparagine N-glycosylation is thought to be essential for the structure and function of glycoproteins^5^. Previous reports implicated ALG13 gene mutations were regarded as a cause of X-linked congenital disorders of glycosylation type I (CDG-I), also known as ALG13-CDG^6,7^. CDG-I, a rare human genetic disorder, characterized by defective glycosylation pathways. Asparagine N-glycosylation is crucial for multiple biological processes and thus CDG patients present highly variable multisystem phenotype that including developmental delay/intellectual disability, muscle hypotonia, seizures, endocrine and coagulation abnormalities, and psychomotor retardation in multiple organ systems^8–11^. Epilepsy is commonly observed in CDG and is often a presenting symptom^8^. In research by Barba et al.^12^, partial seizures were observed in 4 of 17 consecutively observed children with CDG (three females, one male), related testing and analysis indicated CDG-I, two patients were proved with homozygous or compound heterozygous variants through genetic testing. Similarly, in study by Timal et al.^13^, a missense mutation in the X-linked gene ALG13 was found in a child with epilepsy and early death caused by CDG-1.

Previous studies in our laboratory have reported that a wide distribution of ALG13 was observed in the mice brain, particularly in cortex and hippocampus, which is the primary zone closely related to epilepsy^14^. Moreover, our study also shows that ALG13 deficiency increased seizure susceptibility induced by kainic acid or pilocarpine^14^. To our surprise, about 20% of ALG13KO mice with frequent epileptiform convulsion were observed accidentally later. In light of this direct evidence, we intend to study the role of ALG13 in epileptic seizures.

## Results

### ALG13KO mice display spontaneous seizures

**Fig. 1:**
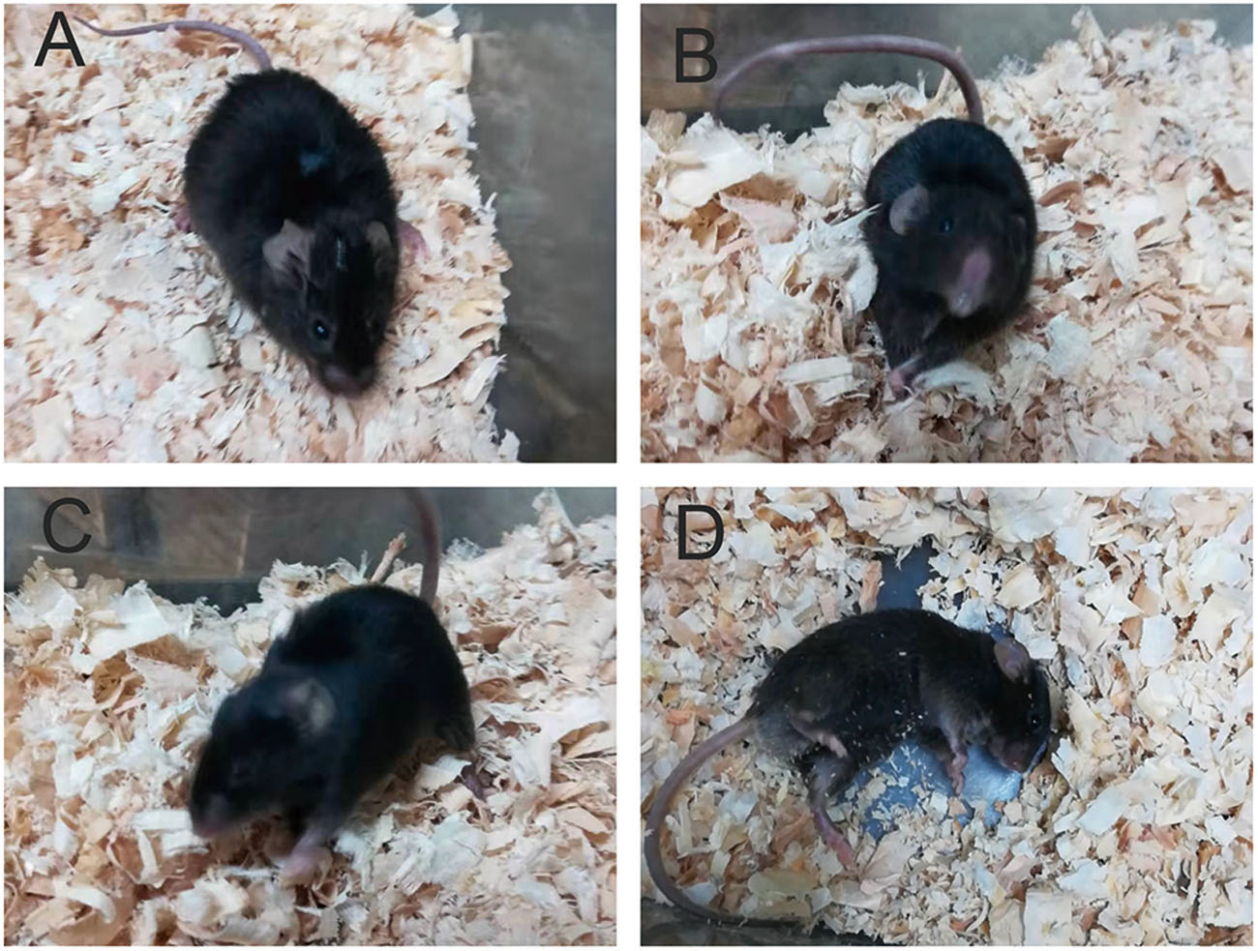
Spontaneous seizures in ALG13KO mice. **a** Onset of the seizure with movement arrest; **b** hypertonic neck and rigidity of the tail; **c** uncontrolled jumping and running; **d** loss of postural, followed by postictal immobility.

There were no obvious difference in the behavior and appearance of ALG13KO mice and wild-type littermates at birth. Around 20% of adult ALG13KO mice began to exhibit frequent spontaneous seizures, female mice particularly mostly, especially during cage changing and handling. Typically, seizures consisted of a behavioral sequence including (i) movement arrest; (ii) repeated clonic jerks, restricted to forelimbs, sometimes unilaterally, followed by a hypertonic neck and tail, and accompanied by loss of posture; and (iii) uncontrolled jumping and running, frequently incontinence and loss of postural equilibrium (Fig. 1). Seizures usually ended after hypertonic postures, during the postictal period, mice were immobile for several seconds. Wild-type littermates never displayed spontaneous epileptic manifestations.

### Video-EEG recording demonstrates epileptic activity in ALG13KO mice

**Fig. 2:**
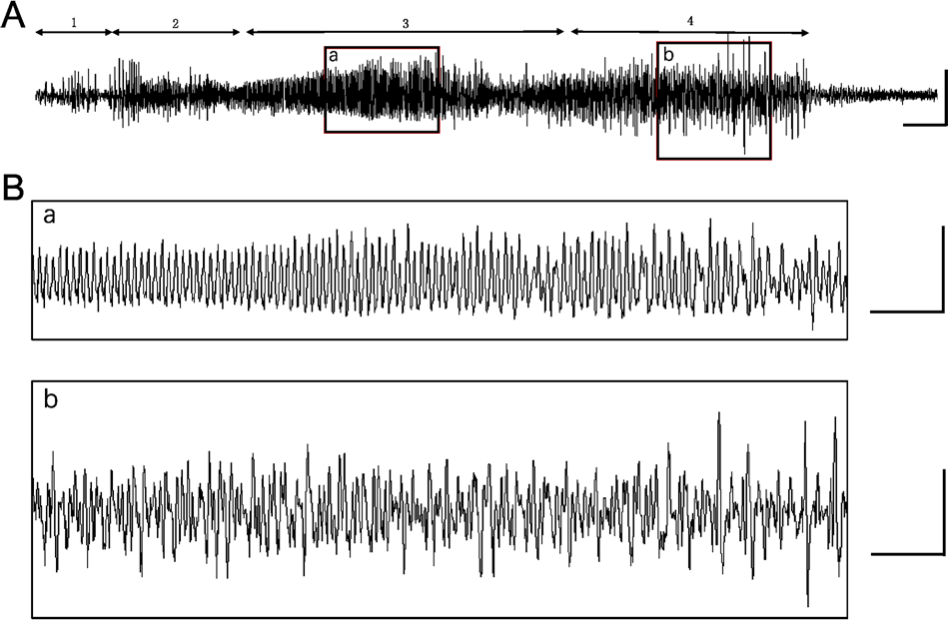
Simultaneous video-EEG recordings of the cortex in ALG13KO mice. **a** Representative traces of EEG activity in ALG13KO mice. Behavioral modifications are correlated with EEG changes: 1 = immobility; 2 = myoclonic jerks; 3 = agitation and wild running; 4 = four-limb hypertonia. Bar, 1 mV, 1 s. **b** (a) Expanded EEG trace shows initial low-amplitude fast activities; (b) spike discharges of increasing amplitude and decreasing frequency. Bar (up), 1 mV, 0.5 s; bar (down), 1 mV, 0.5 s.

**Fig. 3:**
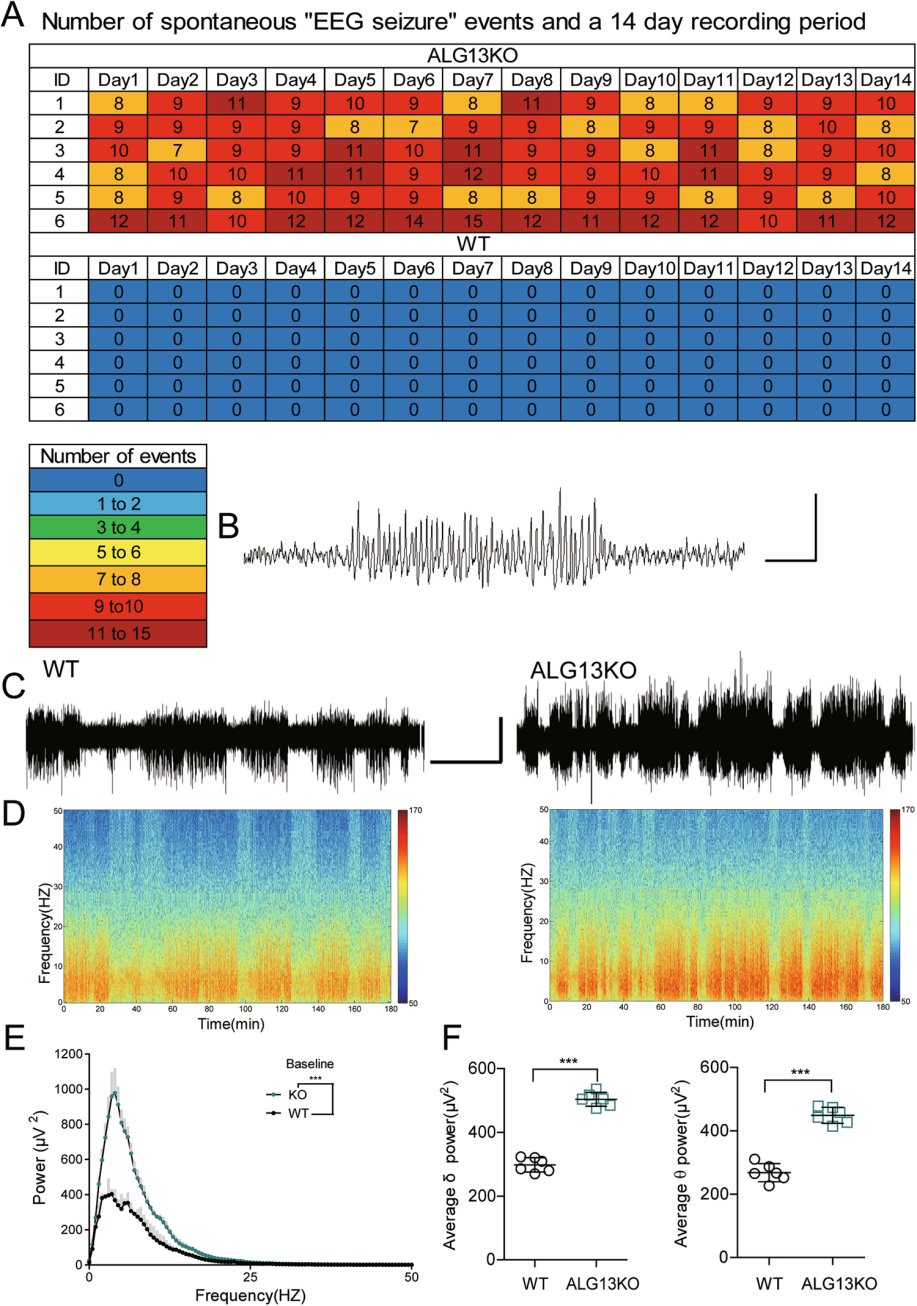
Total number of spontaneous “EEG seizure” events from wild-type and ALG13KO mice without any external intervention (EEGs were recorded for 2 weeks). **a** “EEG seizure” events were detected in C57BL/6J mice implanted with cortical electrodes. Numbers in the first column indicate animal identifiers. Subsequent columns indicate the day during the recording period. Each individual box is color coded according to the legend. Each of the numbers in these boxes indicates the total number of events per day. **b** Example of an event, defined as a high amplitude (>3 times baseline) rhythmic discharge, frequency (>5 Hz), and lasting longer than 5 s. Bar, 1 mV, 1 s. **c** Representative traces of EEG activity in WT and ALG13KO mice without any external intervention. Bar, 1 mV, 15 min. **d** Spectrograms of representative EEG recordings from wild-type and ALG13KO mice during baseline. **e** FFT of EEG recordings comparing ALG13KO and wild-type mice (***P < 0.001, two-way ANOVA test, *n* = 6)). **f** Comparison of average *δ* and *θ* power between wild-type and ALG13KO mice (***P < 0.001, two-sided unpaired *T*-test, *n* = 6).

To explore the phenotype caused by ALG13 mutation, adult ALG13KO mice were studied in simultaneous video and intracranial cortical electroencephalography (EEG) recordings. Ictal epileptic EEG abnormalities were obvious in ALG13KO mice with spontaneous behavioral seizures, and spontaneous seizures were recorded (Fig. 2a). Typically, low-amplitude fast activities; followed by bursts of polyspikes of increasing amplitude and decreasing frequency (Fig. 2b). Moreover, “EEG seizures,” a defined seizure, were evident in ALG13KO mice without spontaneous behavioral seizures (Fig. 3a, b). Epileptic activity was never detected in age-matched wild-type littermates.

We next assessed baseline abnormalities for adult ALG13KO mice in EEG recordings. ALG13KO mice and controls were recorded during 24 h at 1 week. Compared to wild-type littermates, representative spectrograms and fast-Fourier analysis of 3 h of EEG activity indicated increased power of specific frequency bands in ALG13KO mice (Fig. 3c–e). To examine this more depth, we parsed the EEG activity into the following frequency bands: *δ* (0.25–4 Hz), *θ* (4–12 Hz), *α* (12–18 Hz), *β* (18–30 Hz), and low *γ* (30–50 Hz), revealing an elevation in *δ* (WT: 298.1 ± 9.183; ALG13KO: 504.0 ± 8.804) and *θ* (WT: 268.0 ± 11.75; ALG13KO: 449.2 ± 10.46) in ALG13KO mice (Fig. 3f).

### ALG13 deficiency decrease cortical inhibitory synaptic transmission

**Fig. 4:**
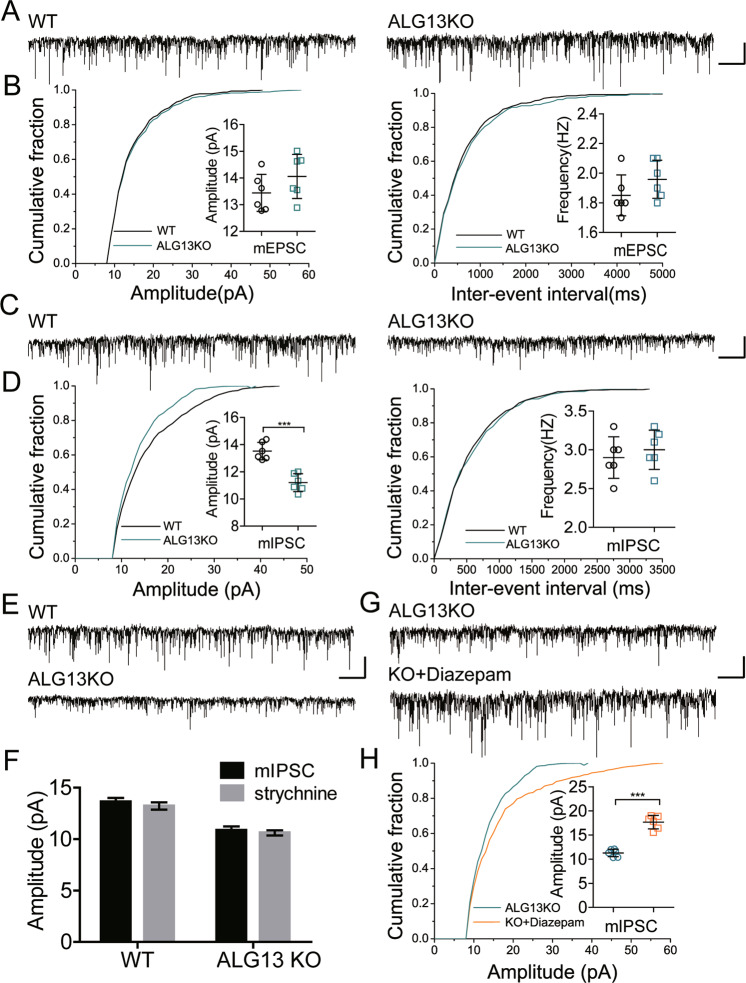
Results of patch-clamp recording. **a** Representative traces of mEPSCs in the cortical pyramidal neurons from p21–30 ALG13KO and wild-type littermate control mice. Bar, 20 pA, 2 s. **b** The amplitude (*P* > 0.05, two-sided unpaired *T*-test, *n* = 6) and the frequency (*P* > 0.05, unpaired *t*-test, *n* = 6) of mEPSCs in the WT and ALG13KO groups. **c** Representative traces of mIPSCs in the cortical pyramidal neurons from p21–30 ALG13KO and wild-type littermate control mice. Bar, 20 pA, 2 s. **d** The amplitude (***P < 0.001, unpaired *t*-test, *n* = 6) and the frequency (*P* > 0.05, two-sided unpaired *T*-test, *n* = 6) of mIPSCs in the WT and ALG13KO groups. **e**, **f** There was not change amplitude of mIPSCs (with strychnine) between ALG13KO group and WT group (*n* = 6). Bar, 20 pA, 2 s. **g**, **h** Treatment with 0.3-mM diazepam increased the amplitude (***P < 0.001, two-sided unpaired *T*-test, *n* = 6) of mIPSC in ALG13KO cortical pyramidal neurons. Bar, 20 pA, 2 s.

To explore the impact of ALG13 on neurotransmission, we recorded miniature excitatory postsynaptic currents (mEPSCs) and miniature inhibitory postsynaptic currents (mIPSCs). Assessments of mEPSC amplitude or frequency did not reveal a significant difference in ALG13KO slices compared to littermate controls (Fig. 4a, b). However, quantification of mIPSC amplitudes revealed a significant decrease between ALG13KO (11.20 ± 0.2668) slices and littermate controls (13.52 ± 0.2613) (***P < 0.001) (Fig. 4c, d). These results show that ALG13 may mainly regulates inhibitory synaptic transmission.

In order to determine whether the effect of ALG13 on mIPSCs involves the effect on glycine current, a set of controlled experiments was performed. The results showed that ALG13 deficiency did not change amplitude of mIPSCs after strychnine was added (Fig. 4e, f). Therefore, ALG13 changes GABAergic transmission without affecting glycine-ergic transmission.

### Treatment of epileptic phenotypes and EEG abnormalities in ALG13KO mice with diazepam

**Fig. 5:**
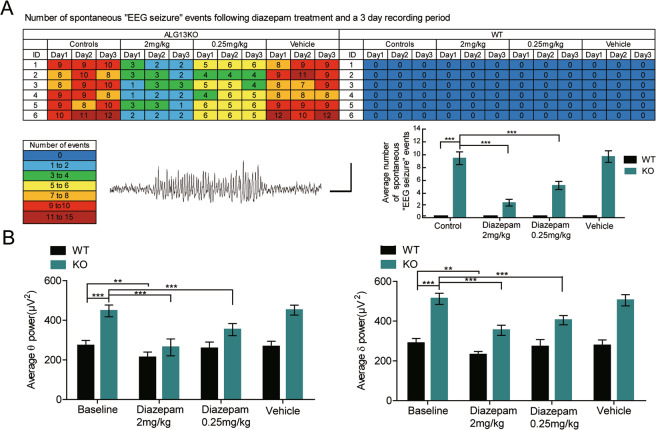
Total number of spontaneous “EEG seizure” events and EEG *δ* and *θ* power from WT and ALG13KO mice following diazepam treatment (2 and 0.25 mg/kg). **a** “EEG seizure” events were detected in ALG13KO mice implanted with cortical electrodes. Numbers in the first column indicate animal identifiers. Subsequent columns indicate the day during the recording period. Each individual box is color coded according to the legend. Each of the numbers in these boxes indicates the total number of events per day (*n* = 6 per group). Example of an event, defined as a high amplitude (>3 times baseline) rhythmic discharge, frequency (>5 Hz), and lasting longer than 5 s. Average number of spontaneous “EEG seizure” events from WT and ALG13KO mice following diazepam treatment (***P < 0.001, treatment of 2-mg/kg diazepam: WT compared with ALG13KO, one-way ANOVA test, *n* = 6; ***P < 0.001, treatment of 0.25-mg/kg diazepam: WT compared with ALG13KO, one-way ANOVA test, *n* = 6) bar, 1 mV, 1 s. **b** Comparison of baseline average *δ* and *θ* power between wild-type and ALG13KO mice and after treatment with 2 and 0.25-mg/kg diazepam (***P < 0.001, baseline: WT compared with ALG13KO, *n* = 6; **p < 0.01, WT: baseline compared with diazepam (2 mg/kg), *n* = 6; *p* > 0.05, baseline compared with diazepam (0.25 mg/kg), *n* = 6; ***P < 0.001, ALG13KO: baseline compared with diazepam (2 mg/kg), *n* = 6; ***P < 0.001, baseline compared with diazepam (0.25 mg/kg), *n* = 6. One-way ANOVA with a post hoc Bonferroni tests was performed.

Given that the epileptic phenotype and EEG abnormalities in ALG13KO mice may emerge from decreased GABAergic inhibitory transmission, we predicted that they could be rescued by enhancing the strength of GABAergic transmission. To test this idea, we tested the effects of diazepam, a positive allosteric modulator of the GABA_A_ receptor, on GABAergic inhibitory transmission in ALG13KO mice cortical slices. As expected, treatment with 0.3-mM diazepam increased mIPSC amplitude (***P < 0.001) (Fig. 4g, h), then we examined the responsiveness of ALG13KO and wild-type mice to diazepam. As expected, both ALG13KO and wild-type mice showed a reduction in epileptic activity following treatment with diazepam (2 mg/kg) as evidenced by a decreased frequency of the “EEG seizure” in the EEG recordings. Due to high doses of diazepam cause sedation, the lower dose of diazepam also was used. We found that increased frequency of the “EEG seizure” and abnormally elevated *δ* and *θ* power in ALG13KO mice, which could be alleviated by treatment with diazepam (0.25 mg/kg) (***P < 0.001), but not in wild-type mice (Fig. 5a, b). These results support our hypothesis that ameliorative epileptic phenotype and EEG abnormalities by treatment with diazepam are related to increased strength of inhibitory transmission.

### ALG13 influences epileptic activity by reducing GABA_A_R α2 subunit gene expression

**Fig. 6:**
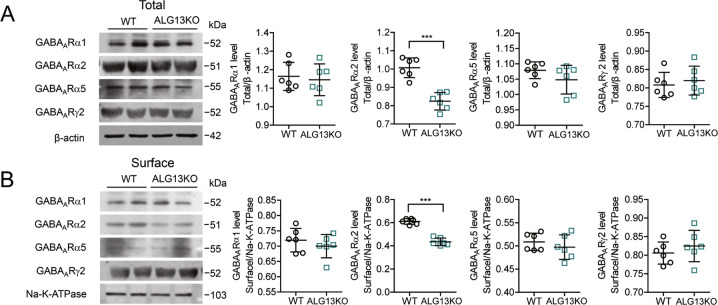
ALG13 affects the total and membrane protein expression of GABA_A_Ra2. **a** Representative images of the total expression levels of GABA_A_Rα1 α2, α5, and γ2 and statistical analyses of the total expression levels of GABA_A_Rα1, α2, α5, and γ2 determined using western blotting analyses (***P < 0.001, expression levels of GABA_A_Rα2: WT compared with ALG13KO, two-sided unpaired *T*-test, *n* = 6). There were no significant differences in the level of total GABA_A_R α1, α5, and γ2 between the WT group and the ALG13KO group. **b** Representative images of the membrane expression levels of GABA_A_Rα1, α2, α5, and γ2 and statistical analyses of the membrane expression levels of GABA_A_Rα1, α2, α5, and γ2 determined using western blotting analyses (***P < 0.001, expression levels of GABA_A_Rα2: WT compared with _A_LG13KO, two-sided unpaired *T*-test, *n* = 6). There were no significant differences in the level of membrane GABA_A_R α1, α5, and γ2 between the WT group and the ALG13KO group.

**Fig. 7:**
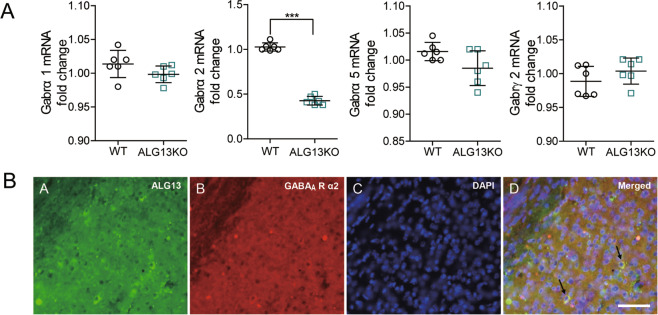
ALG13 affects the mRNA expression of Gabrα2; the cell localization of ALG13 and GABA_A_Rα2. **a** The expression levels of Gabrα2 mRNA was significantly lower in the ALG13KO group than that in the corresponding control (***P < 0.001, expression levels of Gabrα2: WT compared with ALG13KO, two-sided unpaired *T*-test, *n* = 6). There were no significant differences in the level of total Gabrα1, α5, and γ2 mRNA between the WT group and the ALG13KO group. **b** Immunofluorescent triple-staining of ALG13 (green), GABA_A_Rα2 (red), and DAPI (blue) in the cortex, scale bar = 20 µm.

To further investigate the mechanism by which ALG13 affects the inhibitory postsynaptic current in epilepsy, we examined the effect of ALG13 on GABA_A_R regulation. There was no difference in the expression level of total and membrane GABA_A_R α1, α5, and γ2 protein between the ALG13KO group and the WT group (Fig. 6a, b). However, compared with the wild-type group (total: 1.007 ± 0.021; membrane: 0.6108 ± 0.009), the expression of GABA_A_Rα2 total and membrane protein was reduced in the ALG13KO group (total: 0.8243 ± 0.019; membrane: 0.4350 ± 0.013) (***P < 0.001) (Fig. 6a, b). Furthermore, compared with the wild-type group (1.027 ± 0.018), the expression of GABA_A_Rα2 mRNA was significantly reduced in the ALG13KO group (0.4267 ± 0.019) (***P < 0.001) (Fig. 7a). Immunofluorescent result showed probable protein–protein interactions between ALG13 and GABA_A_Rα2 (Fig. 7b) in the cortex of wild-type mice. Taken together, these results suggest that ALG13 affected mRNA expression of Gabrα2 gene, which affected total and surface protein expression of GABA_A_Rα2 in the cortex. ALG13 might affect the expression of membrane protein via interaction with GABA_A_Rα2 or by altering the transcription of GABA_A_Rα2, and further influence mIPSC.

## Discussion

We have characterized the phenotype of ALG13KO mice, showing spontaneous seizures and electroclinical characterization of seizures with video-EEG monitoring and providing details of seizure semiology. Furthermore, we found that loss of ALG13 may reduce the inhibitory synaptic transmission by regulating the transcription of GABA_A_R α2 subunit.

Our previous studies have investigated the distribution and localization of ALG13 in mouse brain, the histological results suggested that ALG13 was highly expressed in the cortex and hippocampus^14^, which are the primary regions of epileptic pathological alterations^15,16^. Immunofluorescent results showed ALG13 to be expressed in neurons but not in astrocytes. These characteristics of ALG13 expression indicates that ALG13 may be involved in epilepsy.

Video-EEG studies on ALG13KO mice confirmed that seizures were often initiated by behavioral immobility, followed by forelimb licking, clonus, succeeding seizures tended to terminate with wild running and tonic–clonic movements. The epileptic phenotype in these animals is similar to postictal behaviors following chemoconvulsant TLE models in animals^17,18^. Interictal EEG activity, with varied discharges initiating as low-voltage fast activities followed by spike and polyspike-wave discharges of increasing amplitude and decreasing frequency, is similar to intracranial EEG recordings of chemoconvulsant TLE models^19–21^. Furthermore, neuropathological changes were found in our previous studies^14^. These changes, which include neuronal cell death, reactive astrogliosis, and aberrant mossy fiber sprouting in the dentate gyrus are typical for human temporal lobe seizures and many animal models of hippocampal seizures^22–24^. Taken together, the results above appear to suggest that ALG13KO mice may be an ideal animal model of temporal lobe epilepsy.

The normal functioning of the cortex depends on a perfect balance between excitatory and inhibitory input, so any disturbance of this balance brings the possibility of uncontrolled hyperexcitability^25,26^. In the present study, deficiency of ALG13 decreased mIPSC amplitude. In general, the amplitude of mIPSC discharge reflects the postsynaptic response to released neurotransmitter, and frequency changes are associated with a presynaptic effect^27^. These results suggest that ALG13 may affect the inhibitory synaptic transmission via postsynaptic receptors.

GABA and glycine are the major inhibitory neurotransmitters in the CNS. Glycine-ergic currents very similar to GABAergic, and GABA_A_R antagonists also act at glycine receptor^28^. Strychnine is an antagonist of glycine, which can selectively inhibits glycine-ergic activity. Our results indicated that ALG13 deficiency did not change amplitude from the first (no strychnine) and second (with strychnine) experiment. Thus, we speculated that ALG13 might influence mIPSCs by regulating postsynaptic GABA_A_Rs.

Benzodiazepine has been widely used to alleviate epileptic seizure^29,30^. Experimental evidence suggested that benzodiazepine can reverse decreased GABAergic tone^31,32^. Our results indicate that treatment with low nonsedating dose benzodiazepine increase inhibitory neurotransmission, which could be a potential pharmacological intervention for patients with mutation in the ALG13.

Many GABA_A_ receptors are composed of two α subunits, two β subunits and one γ subunit. The binding site for benzodiazepines is made up with one of the α subunits (α1, α2, α3, or α5) and a γ subunit (typically the γ2 subunit)^33,34^. The number of GABA_A_ receptors in the postsynaptic membrane directly controls the efficacy of GABAergic synaptic transmission^35^. We investigated the expression of the GABA_A_Rs in ALG13KO and wild-type mice. There was no difference in the expression level of total or surface GABA_A_Rα1, α5, and γ2 protein in the cortex between the ALG13KO groups and the WT groups. However, deficiency of ALG13 reduced the expression of GABA_A_Rα2 membrane protein. Meanwhile, we find that total GABA_A_Rα2 protein displayed the same change in the cortex. This finding suggests that the synthesis of GABA_A_Rα2 protein may change. To further test this hypothesis, we used real-time PCR to analyze transcription of mRNA. The results indicated that deficiency of ALG13 affected the transcription level of GABA_A_Rα2. In addition, In our study, Immunofluorescent revealed that ALG13 interacts with GABA_A_Rα2.

In conclusion, our findings demonstrate that mutations in ALG13 can led to epilepsy and alleviate GABA_A_R-mediated postsynaptic transmission. Further, ALG13 regulated the expression of GABA_A_Rα2 total and membrane proteins possibly through affecting the transcription of GABA_A_Rα2 or via interaction with GABA_A_Rα2. These may be major contributor to epileptogenesis. However, the mechanism whereby ALG13 regulates the GABA_A_ receptor still needs to be explored further and in greater detail.

## Materials and methods

### Experimental animals

ALG13 KO mice created with a C57BL/6J background were provided by Y.B. (Shanghai Jiaotong University, Shanghai, China). Gene knockout mice were generated by deleting five nucleotides of the fourth exon of the ALG13 gene using CRISPR-Cas9 systems. Genotyping of ALG13KO mice as previously described in our study (Gao et al.^14^). All mice were kept in a specific-pathogen-free environment under standard conditions with a 12-h light/dark cycle and controlled temperature at 22 °C with food and water available ad libitum. Animals were randomly assigned to different experimental groups. 10–12-week-old male mice were used in all experiments except the patch-clamp experiment. All procedures used on mice were approved by the Commission of Ningxia Medical University for Ethics of Experiments on Animals and were in accordance with the National Institutes of Health Guide for the Care and Use of Laboratory Animals.

### Electroencephalography (EEG)

Surgery and recording 10–12-week-old male mice were used for EEG studies. Prefabricated light-weight EEG electrodes (2-channel) were surgically implanted under isofluorane anesthesia (3–5% for induction, 1–2% for maintenance), placing the electrodes leads into the subdural space. Electrodes were anchored to the skull and over the left frontal cortex (reference electrode) and the left and right parietal cortices as described using stainless steel screws and acrylic cement. All EEG recordings were carried out at least 1 week after surgery on freely moving mice in a recording chamber. Digital EEG activity and videos of their locomotor activity were recorded with SleepSign v.3.0 (Kissei Comtec, Japan). A spontaneous “EEG seizure” event was defined as a high amplitude (>3 times baseline) rhythmic discharge, frequency (>5 Hz), and lasting longer than 5 s^36^. Spectral power was obtained by subjecting the recordings to a fast-Fourier transform using SleepSign. Results were quantified using SleepSign and Matlab.

### Diazepam intervention

Four sets of identical trials were performed at 1-week intervals with the same groups of mice. In the first trial, we performed the long-term continuous EEG monitoring without any treatment. In a subsequent trial, the same tests were performed after intraperitoneal injection of 2-mg/kg clonazepam. In the third trial, the same tests were performed after intraperitoneal injection of 0.25-mg/kg clonazepam. In the last trial, the tests were performed after intraperitoneal injection of vehicle.

### Whole-cell patch-clamp recordings

Mice at postnatal day 21 (P21) to P30 were anesthetized with isoflurane and quickly decapitated, Coronal slices (400-μm thickness) were prepared (Leica VT1000S) in a cold sterile slice solution (mM: 124 NaCl, 3 KCl, 2 CaCl_2_, 2.5 MgSO_4_, 26 NaHCO_3_, 1.25 NaH_2_PO_4_, 10 glucose, (pH 7.4) bubbled with 95% O_2_/5% CO_2_) and transferred into an incubation chamber filled with prewarmed (22–24 °C) oxygenated artificial cerebrospinal fluid of the following composition (mM: 124 NaCl, 3 KCl, 2 CaCl_2_, 2.5 MgSO_4_, 26 NaHCO_3_, 1.25 NaH_2_PO_4_, 10 glucose, bubbled with 95% O_2_/5% CO_2_) for a recovery period of 1 h before recording.

For the mIPSCs recording, whole-cell currents were recorded from the layer 2/3 of the neocortex pyramidal cells, pipette electrodes (3–5 MΩ) pulled from borosilicate glass (Sutter), and filled with intracellular solution (mM: 100 CsCl, 1 MgCl_2_, 1 EGTA, 10 HEPES, 12 phosphocreatine, 30 N-methyl-d-glucamine (NMG), 5 Mg-ATP, and 0.5 Na_3_GTP; pH = 7.2 with CsOH). The membrane was voltage-clamped at −70 mV, mIPSCs were recorded in the presence of tetrodotoxin (TTX) (1 μM; to block sodium current), DL-2-amino-5-phosphonopentanoic acid (AP-V) (50 μM, MCE; to block NMDA receptors), and 6, 7-dinitroquinoxaline-2, 3(1H, 4H)-dione (20 μM, MCE; to block AMPA receptors). A 5-min period for stabilization after obtaining recording was recorded.

For the mEPSCs recording, whole-cell currents were recorded from the layer 2/3 of the neocortex pyramidal cells, pipette electrodes (3–5MΩ) pulled from borosilicate glass (Sutter), and filled with intracellular solution (mM: 130 CsMeS, 10 CsCl, 1 MgCl_2_, 4 NaCl, 1 EGTA, 10 HEPES, 12 phosphocreatine, 5 NMG, 5 Mg-ATP, and 0.5 Na_3_GTP; pH = 7.2 with CsOH). Membrane potential was held at −70 mV, TTX (1 μM) and picrotoxin (50 μM, MCE; to block GABA_A_ receptors) were added to the bath solution. A 5-min period for stabilization after obtaining recording was recorded.

To determine the ability of ALG13 in altering the GABAergic synaptic current or glycine-ergic current, strychnine (0.5 mM, MCE) was infused into ACSF to selectively inhibit glycine-ergic activity. mIPSCs were recorded in the pyramidal neurons after strychnine administration.

After the baseline data were recorded, diazepam (0.3 mM, sigma) was added to the perfusate. All recordings were conducted in standard ACSF with 0.1% DMSO. Signals were obtained from Patch-Clamp Amplifier (HEKA EPC10 double USB, German), filtered at 2 kHz and digitized at 10 kHz, recorded by PatchMaster (HEKA, German). The data were discarded if the series resistance fluctuated by more than 25% of the initial value. Mini Analysis 6.0.1 was used to analyze the recorded trace. Origin Pro 8.0 software was applied to convert exported data to the graphical format.

### Western blotting

The cortex was excised, weighed, and washed in phosphate-buffered saline and subsequently homogenized on ice using a Bullet Blender (Next Advance, Inc., Troy, NY, USA) in lysis buffer consisting of phosphatase inhibitors, protease inhibitor, and phenylmethylsulfonyl fluoride. Then, the homogenates were centrifuged at 12000 *g* for 15 min at 4 °C, and the total protein was collected with the supernatant. The membrane proteins were extracted according to the instructions for the Membrane Protein Extraction Kit (KeyGen, Nanjing, China). The homogenate was incubated at 4 °C for 10 min and centrifuged at 12,000 rpm at 4 °C for 5 min. The precipitate obtained by centrifugation was taken, 200 μl of cold extraction buffer was added, vortexed, and mixed for 30 s, and then placed on ice for 5 min, repeated five times. The homogenate was centrifuged at 12,000 rpm at 4 °C for 10 min, and supernatant fractions were collected. The protein concentrations were then determined using a bicinchoninic acid kit (BCA kit; KeyGen, Nanjing, China). Before boiling at 100 °C for 6 min to denature, 80 μg of protein sample was added into loading buffer. Protein samples were separated by 10% SDS-polyacrylamide gel electrophoresis gels and electrotransferred onto 0.45-μm polyvinylidene difluoride membranes (Millipore, Billerica, MA, USA). The membranes were blocked for 1 h with 5% skim milk powder in TBST at room temperature and then incubated with rabbit anti-GABA_A_Rα1 (1:6000 Abcam, ab33299), anti-GABA_A_Rα2 (1:1000 Abcam, ab153980), anti-GABA_A_Rα5 (1:1000 Abcam, ab10098), anti-GABA_A_Rγ2 (1:500 Abcam, ab87328), anti-Na-K-ATPase (1:1000, Cell Signaling Technology, MA, USA), β-actin (1:1000, ZSGB-bio, ZM-0001) antibodies overnight at 4 °C. The blots were washed three times and incubated for 1 h with horseradish peroxidase-conjugated anti-rabbit secondary antibodies (1:5000) in TBST with 5% milk. The blots were then washed in TBST, and bands were visualized using an enhanced chemiluminescence reagent (Thermo, Marina, CA, USA) or the Odyssey CLX instrument system (LI-COR, USA). Subsequently, the chemiluminescence method was employed and the relative target protein levels were evaluated after X-ray film exposure. Results were quantified using Image J.

### Real-time reverse transcription polymerase chain reaction (RT-PCR)

The total RNA of the cortices was isolated with the TRIzol solution (100-mg tissue/ml; Invitrogen, Carlsbad, CA, USA), according to the manufacturer’s protocol. The OD260/280 value of extracted RNA sample was detected utilizing a spectrophotometer. Next, cDNA was synthetized using Revert Aid First Strand cDNA Synthesis Kit (Thermo Scientific, K1621). The gene sequences of the GABAAR subunits were designed from the National Center for Biotechnology Information database.

The primer sequences were as follows:

Gabrα1 (mouse) forward: 5′-GCGTATCACAGAGGATGGCACTC-3′

Gabrα1 (mouse) reverse: 5′-TCTTCTGCTACAACCACTGAACGG-3′

Gabrα2 (mouse) forward: 5′-TGCGGTAGCTGTTGCCAACTATG-3′

Gabrα2 (mouse) reverse: 5′-CTCTGGCTTCTTGTTCGGTTCTGG-3

Gabrα5 (mouse) forward: 5′-ACACCATGCGTCTGACAATCTCTG-3′

Gabrα5 (mouse) reverse: 5′-GCCATCTTCTGCCACCACCAC-3

Gabrγ2 (mouse) forward: 5′-ACACCATGCGTCTGACAATCTCTG-3′

Gabrγ2 (mouse) reverse: 5′- GCCATCTTCTGCCACCACCAC-3

GAPDH (mouse) forward: 5′-TTGTCATGGGAGTGAACGAGA-3′

GAPDH (mouse) reverse: 5′-CAGGCAGTTGGTGGTACAGG-3′

The synthesized cDNA was used for RT-PCR. The cDNA was amplified using a one-step qPCR kit (SYBR Green, Bestar, Shanghai, China). The RT-qPCR reaction conditions were set as follows: predenaturation at 95 °C for 2 min, a cycle of 40 times of denaturation at 95 °C for 10 s, annealing at 58 °C for 30 s, extension at 72 °C for 15 s with GAPDH used as the internal reference. Relative mRNA levels of each target gene was determined by the average cycle threshold value (Ct) followed by normalization for the GAPDH. The fold changes of target gene were quantified by using formula: ΔCt (target) = Ct (target gene) − Ct (GAPDH) and ΔΔCt = ΔCt (experimental group) − ΔCt (control group); 2^−ΔΔCt^ was used to evaluate the relative expression of the target gene.

### Experimental design and statistical analysis

A sample size of 6 was arbitrarily chosen for first EEG and whole-cell patch-clamp study. Because there was a statistically significant difference using *n* = 6, we chose to continue using this sample size for all our studies. According to whether the samples exhibited normal distributions and equal variances (determined by the one-sample Kolmogorov–Smirnov test and Levene’s test), the experimental results were statistically assessed using parametric or nonparametric tests. All data are expressed as mean ± SD, and comparisons between two groups were performed using unpaired Student’s two-tailed *t* test. One-way analysis of variance (ANOVA) with a post hoc Bonferroni test was used to measure differences between multiple groups considering one fixed factor. Two-way ANOVA with a post hoc Bonferroni test was used to measure differences between two groups comparisons considering two fixed factors. *P* value < 0.05 was considered to be statistically significant. SPSS 23.0 and GraphPad Prism 5.0 software were used for statistical analyses and graphing, respectively.

## References

[1] Noebels JL (2003). The biology of epilepsy genes. Annu. Rev. Neurosci..

[2] Steinlein OK (2004). Genetic mechanisms that underlie epilepsy. Nat. Rev. Neurosci..

[3] Averbeck N, Gao XD, Nishimura S, Dean N (2008). Alg13p, the catalytic subunit of the endoplasmic reticulum UDP-GlcNAc glycosyltransferase, is a target for proteasomal degradation. Mol. Biol. Cell.

[4] Gao X-D, Moriyama S, Miura N, Dean N, Nishimura S-I (2008). Interaction between the C Termini of Alg13 and Alg14 mediates formation of the active UDP-N-acetylglucosamine transferase complex. J. Biol. Chem..

[5] Brasil, S. et al. CDG therapies: from bench to bedside. *Int. J. Mol. Sci.* **19**, 2018. 10.3390/ijms19051304 PMC5983582 29702557

[6] Bissar-Tadmouri N (2014). X chromosome exome sequencing reveals a novel ALG13 mutation in a nonsyndromic intellectual disability family with multiple affected male siblings. Am. J. Med. Genet. A.

[7] de Ligt J (2012). Diagnostic exome sequencing in persons with severe intellectual disability. N. Engl. J. Med..

[8] Freeze HH, Eklund EA, Ng BG, Patterson MC (2015). Neurological aspects of human glycosylation disorders. Annu. Rev. Neurosci..

[9] Ferreira CR (2018). Recognizable phenotypes in CDG. J. Inherit. Metab. Dis..

[10] Galama WH, Verhaagen-van DASLJ, Lefeber DJ, Feenstra I, Verrips A (2018). ALG13-CDG with infantile spasms in a male patient due to a de novo ALG13 gene mutation. JIMD Rep..

[11] Verheijen J, Tahata S, Kozicz T, Witters P, Morava E (2020). Therapeutic approaches in congenital disorders of glycosylation (CDG) involving N-linked glycosylation: an update. Genet. Med..

[12] Barba C (2016). Congenital disorders of glycosylation presenting as epileptic encephalopathy with migrating partial seizures in infancy. Dev. Med. Child Neurol..

[13] Timal S (2012). Gene identification in the congenital disorders of glycosylation type I by whole-exome sequencing. Hum. Mol. Genet..

[14] Gao P (2019). ALG13 deficiency associated with increased seizure susceptibility and severity. Neuroscience.

[15] Bronen RA (1991). Imaging findings in hippocampal sclerosis: correlation with pathology. AJNR Am. J. Neuroradiol..

[16] Wan Q (1997). Recruitment of functional GABA(A) receptors to postsynaptic domains by insulin. Nature.

[17] Mouri G (2008). Unilateral hippocampal CA3-predominant damage and short latency epileptogenesis after intra-amygdala microinjection of kainic acid in mice. Brain Res..

[18] Pernot F (2011). Inflammatory changes during epileptogenesis and spontaneous seizures in a mouse model of mesiotemporal lobe epilepsy. Epilepsia.

[19] French ED, Aldinio C, Schwarcz R (1982). Intrahippocampal kainic acid, seizures and local neuronal degeneration: relationships assessed in unanesthetized rats. Neuroscience.

[20] Medvedev A, Mackenzie L, Hiscock JJ, Willoughby JO (2000). Kainic acid induces distinct types of epileptiform discharge with differential involvement of hippocampus and neocortex. Brain Res. Bull..

[21] Navarro V (2002). Seizure anticipation in human neocortical partial epilepsy. Brain: J. Neurol..

[22] Dudek FE, Sutula TP (2007). Epileptogenesis in the dentate gyrus: a critical perspective. Prog. Brain Res.

[23] Pekny M (2016). Astrocytes: a central element in neurological diseases. Acta Neuropathol..

[24] Sofroniew MV (2014). Astrogliosis. Cold Spring Harb. Perspect. Biol..

[25] Bonansco C, Fuenzalida M (2016). Plasticity of hippocampal excitatory-inhibitory balance: missing the synaptic control in the epileptic brain. Neural Plast..

[26] Rubenstein JL, Merzenich MM (2003). Model of autism: increased ratio of excitation/inhibition in key neural systems. Genes Brain Behav..

[27] Oberlander JG, Woolley CS (2016). 17Beta-estradiol acutely potentiates glutamatergic synaptic transmission in the hippocampus through distinct mechanisms in males and females. J. Neurosci..

[28] Mori M, Gahwiler BH, Gerber U (2002). Beta-alanine and taurine as endogenous agonists at glycine receptors in rat hippocampus in vitro. J. Physiol..

[29] Burman RJ (2019). Excitatory GABAergic signalling is associated with benzodiazepine resistance in status epilepticus. Brain.

[30] Goodkin HP, Kapur J (2009). The impact of diazepam’s discovery on the treatment and understanding of status epilepticus. Epilepsia.

[31] Han S (2012). Autistic-like behaviour in Scn1a+/− mice and rescue by enhanced GABA-mediated neurotransmission. Nature.

[32] Jacob TC (2012). Benzodiazepine treatment induces subtype-specific changes in GABA(A) receptor trafficking and decreases synaptic inhibition. Proc. Natl Acad. Sci. U. S. A..

[33] Olsen RW, Sieghart W (2008). International Union of Pharmacology. LXX. Subtypes of gamma-aminobutyric acid(A) receptors: classification on the basis of subunit composition, pharmacology, and function. Update Pharm. Rev..

[34] Rudolph U, Knoflach F (2011). Beyond classical benzodiazepines: novel therapeutic potential of GABAA receptor subtypes. Nat. Rev. Drug Discov..

[35] Luscher B, Fuchs T, Kilpatrick CL (2011). GABAA receptor trafficking-mediated plasticity of inhibitory synapses. Neuron.

[36] Jimenez-Mateos EM (2012). Silencing microRNA-134 produces neuroprotective and prolonged seizure-suppressive effects. Nat. Med..

